# Supplementary material for: Charge Transport Enhancement in BiVO4 Photoanode for Efficient Solar Water Oxidation
Source: Materials (Basel). 2023 Apr 27;16(9):3414. doi: 10.3390/ma16093414 (PMC10180425; doi:10.3390/ma16093414)
Supplement: Supplementary file 1 [file materials-16-03414-s001.zip › materials-2324972-supplementary.pdf]

# Supplementary Materials

# Charge Transport Enhancement in $BiVO_4$ Photoanode for Efficient Solar Water Oxidation

**Zhidong Li, Zhibin Xie, Weibang Li, Hafiz Sartaj Aziz, Muhammad Abbas, Zhuanghao Zheng, Zhenghua Su, Ping Fan, Shuo Chen * and Guangxing Liang ***

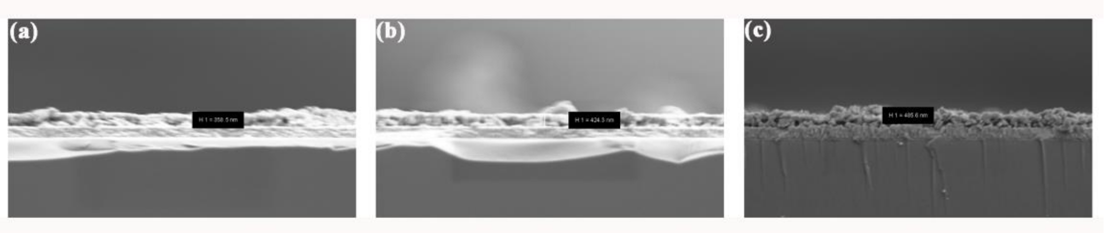


Figure S1. Cross-sectional SEM images of the (a) $BiVO_4$-1, (b) $BiVO_4$-2, and (c) $BiVO_4$-3 film.

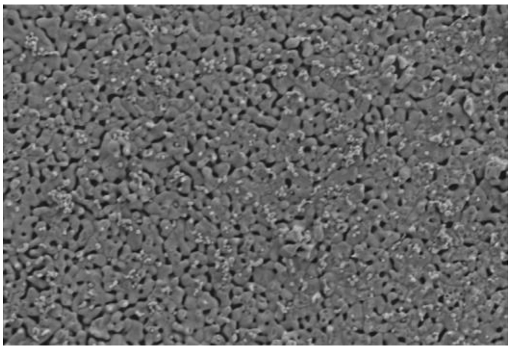

Figure S2. Top-view SEM image of the $BiVO_4$/CoPi photoanode.

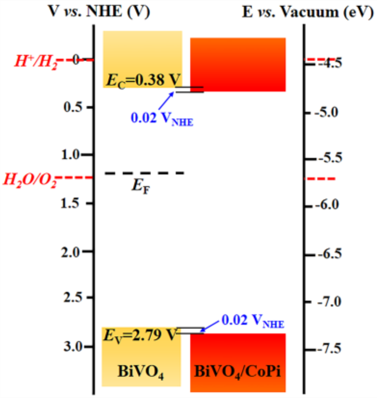


Figure S3. The energy levels schematic diagram of the $BiVO_4$ and $BiVO_4$/CoPi photoanodes.

**Supplementary Note S1.** UPS analysis

The work function (Φ) and Fermi level ($E_F$) can be calculated by the following equations:

$$\Phi = h\nu - E_{cut-off}$$

$$\Phi = E_0 - E_F$$

Where hν is 21.22 eV (He I source), $E_{cut\text{-}off}$ is the secondary electron cut-off edge, and $E_0$ is 0 eV vs. vacuum.
